# Operations for Cataract, upon Two Brothers, Who Were Deaf, Dumb, and Blind

**Published:** 1855-10

**Authors:** George Dock

**Affiliations:** Harrisburg, Pa.


*Operations for Cataract, upon two brothers, who were deaf, dumb, and blind.* By George Dock, M. D., of Harrisburg, Pa.

In the dusty arena of the medical profession, cases occasionally come under the notice of the practitioner, a short history of which, from their rareness and peculiarity, may not be unacceptable for the perusal of his professional brethren. Such are the cases, the history of which I now offer, should you deem them worthy of a place in your valuable Journal.

In the spring of 1853, a gentleman named James Devor, of Perry county, Pa., came to my office to consult me in relation to the case of his two brothers, and to solicit my services on their behalf. His account of them was as follows: "They were both born deaf and dumb; at a proper age were sent to Philadelphia to be educated at the Asylum for the Deaf and Dumb. After having completed their course of instruction at that place, they returned home (to Franklin Co.) fine, active, intelligent boys. Their names are William and John. William is 50, John 39 years of age. At the age of 35 William became blind, and at 28 John lost his sight, so that to the present time they have been respectively blind fifteen and eleven years."

Upon hearing the above account of them, I was, at first, inclined to look upon them as hopeless cases, but as he insisted that I should visit them, I consented to do so. On the appointed day I took the cars, and in a few hours arrived at their house. It was then about twilight, and upon entering the room I found the two brothers sitting side by side, like two statues, perfectly unconscious of everything around them, and only satisfying themselves of each other's presence by occasionally extending the hand and so feeling each other. By the way, they were almost inseparable, the one manifesting signs of uneasiness and discontent whenever he would find the other absent from him. Being curious to learn their method of communication with each other and the rest of the family, a sister took one of them by the hand and very dexterously manœuvred his fingers in the language of the dumb alphabet, thus informing him of my presence and the object of my visit. His sad countenance lit up with a gleam of satisfaction and hope, and he immediately took the hand of his brother, and, in like manner, communicated the intelligence to him. A more interesting sight I think I never witnessed. There they sat, two fine looking, intelligent men, cut off from all the avenues of knowledge of the external world, with nought left them but the power of thought, and that, apparently, the greater part of the time, concentrated upon the gloomy consciousness of their sad lot. Their privations were almost greater than in the case of the unfortunate Laura Bridgman, from the fact of their having had the enjoyment of vision long previous to the loss of it, thus causing them to feel more keenly the present deprivation of it. John seemed somewhat cheerful at times, and was in the enjoyment of good physical health, but the elder brother was more despondent, his mind was irritable, his features were haggard and care-worn, his general health impaired, and his body and limbs becoming emaciated. Long confinement and the want of physical exercise shewed their marks upon him, and had considerably shattered his constitution. Unable to make any satisfactory examination of their eyes that night, I retired; wondering whether it should be my privilege to be, in the hands of Providence, the instrument by which they might be released from their prison of darkness. Upon going down stairs in the morning, I found them both in their accustomed seats, anxiously awaiting me. Upon an examination of their eyes I was overjoyed at finding John's vision obstructed by a clean, pretty firm, pearly cataract of each eye: iris healthy, and retina evidently possessing good tone and sensibility. I then examined William's, but here I was less encouraged: there was cataract also of both eyes, but I found at a glance, that the retina had, from long inactivity and deprivation of the stimulus of light, become partially amaurotic: the pupils largely dilated and responding but little to the changes of light. I feared the mere removal of the cataract would be of little avail, but, as he had nothing to lose, he insisted that I should operate on him also, if I concluded to do so upon his brother. I dropped a solution of atrophine in one eye of each of them, and in half an hour they were both prepared for operation. I took John first, and with a very delicate, slightly curved needle, performed the operation of reclination: succeeding handsomely in lodging the lens and capsule gently into the desired place, leaving a clear, black, and beautiful pupil. I had scarcely withdrawn my instrument when he gave signs of light having poured into his eye, and of his great delight and encouragement as to the entire restoration of his long lost sight.

After disposing of him properly, I operated upon William in the same manner as in John's case, and also succeeded in removing the entire cataract, leaving a large, clear, black pupil, but the light, when let into the eye, produced very little effect upon it, and he appeared a good deal disheartened when he found the operation was finished and there was but little change in his vision.

I returned home, and in a couple of weeks—having received several letters from Dr. Crawford, informing me of their favorable condition and improvement, and great desire to have the other eyes operated upon—I visited them again and found their eyes entirely free from inflammation, and, in John's case, vision good; in William's, a slight improvement, but still imperfect. I now operated upon them again, with equal facility as upon the first occasion, leaving them in charge of Dr. Crawford.

About one month after the last operation had been performed, I received a very encouraging letter from the brother who had employed me, inviting me to pay them a visit. I did so, and found them in the following condition: John was standing in the lane when I approached the house, and as soon as I alighted from the carriage he ran to meet me, grasped me by the hand and made all kinds of demonstrations of joy and gratitude; pointed away down the beautiful valley, to houses and other objects a mile distant from where we stood; made motions that he could shoot with a gun, and go out hunting over the hills. We then went into the house, and I took my pencil and wrote several questions on a bit of paper and handed it to him; he put on a pair of cataract glasses that I had sent to him, and after reading off rapidly what I had written, he took my pencil and wrote his answers to them in quite a good hand writing. He told me his sight was perfectly restored, and as strong as it had ever been before he became blind.

I then asked for his brother William, and was told he was out in the garden, where he was in the habit of sauntering about for exercise. He was brought into the house, and upon examination of his eyes I found some improvement; he could see well enough to walk about with freedom, but could not discern small objects nor distinguish the countenances of persons. The pupils were unobstructed and clear, but were still too large and indolent. I prescribed for him a course of tonic treatment of some of the vegetable bitters, and iron and daily exercise in the open air; the result of which, I had the pleasure of learning some weeks afterward, was great improvement of his general health, with a gradual and encouraging amendment of his vision.

Out of nearly sixty cases that I have operated upon, I know of none which enlisted my sympathy to a greater degree, or the history of which was more interesting or the result of my humble efforts more gratifying.

